# Understanding the essential components and effectiveness of pre-assessment counselling (PAC) in providing a timely diagnosis according to NHS clinicians

**DOI:** 10.1177/14713012251345928

**Published:** 2025-05-26

**Authors:** Marie Janes, Anna Buckell, Bethany A Jones, Miriam Sang-Ah Park, Stephen P Badham

**Affiliations:** 6122 Nottingham Trent University, UK; 5314 Nottinghamshire Healthcare NHS Foundation Trust, UK; Memory Assessment Service, Lings Bar Hospital, Gamston, UK; 6122 Nottingham Trent University, UK

**Keywords:** dementia, qualitative research, pre-assessment counselling, pre-diagnosis counselling, healthcare professionals, Alzheimer’s disease, early-onset dementia, carers, caregivers

## Abstract

This qualitative study explores the significance of Pre-Assessment Counselling (PAC) in supporting timely diagnoses for people with dementia from the perspectives of clinicians. Reflexive thematic analysis was employed to analyse in-depth interviews with a multidisciplinary team of clinicians specialising in dementia care. Three themes were identified: (1) The centrality of people with dementia in their diagnosis journey, (2) The importance of candid conversations in building therapeutic alliances, and (3) Recognising people with dementia are more than their diagnoses. These themes elucidate the multifaceted aspects of PAC and its implications for well-being and engagement in dementia care. The findings underscore the significance of timely diagnoses for the well-being of people with dementia while highlighting the nuanced nature of diagnosis delivery. Moreover, they emphasise the importance of empowering people with dementia in decision-making processes and fostering resilience through comprehensive support. The clinical and research implications of PAC implementation in dementia care are discussed.

## Introduction

Dementia presents a significant challenge, triggering a cascade of emotional, psychological, and financial pressures for individuals, families, and society (Livingston et al., 2020). Approximately 850,000 people in the United Kingdom (UK) have dementia, though it is likely higher when including those without a formal diagnosis (Ford et al., 2019). Underdiagnosis has been associated with social and self-stigma (Nguyen & Li, 2020), inability of people with impaired cognition to recognise symptoms (Harwood et al., 2000), general practitioners (GPs) lacking confidence in diagnosing dementia (Phillips et al., 2012), fear held by people with impaired cognition of the consequence of diagnosis (Page et al., 2019), and a lack of culturally appropriate diagnostic tools (Nielsen & Jørgensen, 2020). The projection is that by 2051, more than two million people in the UK will be living with dementia. Therefore, it is one of Western society’s most significant public health challenges (Skov et al., 2022).

Many people who perceive they have symptoms of dementia will confide in family; however, for some, the fear of dementia is so great that it can deter them from seeking professional advice (Yun & Maxfield, 2020). Avoidance of professional help-seeking in the early stages of dementia is problematic as the literature suggests that both people with dementia and those who care for them benefit most from early diagnosis (Koch et al., 2010). Such benefits include enabling carers the opportunity to adapt to the changes that characterise dementia and to their newfound role, as well as the ability to access interventions to protect well-being and enable self-efficacy of people with dementia (De Vugt & Verhey, 2013).

One potential way to increase help-seeking and potentially an early diagnosis (Price et al., 2011) is to offer pre-assessment counselling (PAC) for dementia (La Fontaine et al., 2014). PAC explains what to expect from the diagnosis process, including possible outcomes. Exploring these implications enables a person with dementia to make an informed decision on whether to pursue an assessment (La Fontaine et al., 2014). Placing the person with dementia at the centre of this decision is essential given the significant social and psychological adjustment required upon receiving a diagnosis of dementia for both the person with dementia and their families (Lecouturier et al., 2008). PAC also provides opportunities to tackle stigma and explore possible fears which may be driven by underlying abilities such as acknowledged cognitive changes; levels of fear are often correlated with the level of problems, highlighting the importance of exploration and discussion (La Fontaine et al., 2014). Research indicates that anxiety towards a potential dementia diagnosis is decreased when people with cognitive deficits are provided with informal feedback prior to formal disclosure, as conversations around potential diagnoses are considered helpful by alleviating shock should dementia be confirmed (Carpenter et al., 2008).

### The current study

As clinical research advances, findings should be refined to improve outcomes for people with dementia. Pre-assessment counselling (PAC) is recognised as beneficial in dementia care (La Fontaine et al., 2014). Yet, a lack of empirical evidence on its mechanisms and effectiveness limits the development of standardised protocols. This study aims to explore experiential and contextual factors surrounding PAC delivery through semi-structured interviews with UK clinicians who provide PAC for dementia. Using Reflexive Thematic Analysis (Braun & Clarke, 2022), it seeks to uncover themes related to PAC’s role in the timely diagnosis process, offering a qualitative understanding of the challenges and facilitators in its implementation and addressing a knowledge gap to support the standardisation and inclusion of PAC in dementia care (Navarrete, 2009).

## Method

### Setting

The setting was a National Health Service (NHS) trust. NHS Trusts are subject to legislation depending on the country within which they are located in the UK. This study liaised with the Early Intervention Dementia Service team based within a West Midlands NHS Trust in England. This service was selected because the clinicians within this team conducted what is internally known as PAC during the initial appointment between the person with cognitive difficulties and those they bring for support. In the UK, government-funded care is organised into different levels. Primary care is often the first point of contact for people needing healthcare. It is provided by professionals such as general practitioners (GPs) and secondary care services, including PAC, who generally need a referral from a GP. Therefore, GPs are often the first clinicians for individuals experiencing cognitive decline, and their families approach them for support and answers regarding symptoms (Phillipson et al., 2015). In the UK, GPs commonly conduct initial assessments and refer suspected dementia cases to memory assessment services or clinics staffed by specialist dementia clinicians, such as psychologists, nurses, psychiatrists, and occupational therapists. Following referral, a clinical assessment typically precedes cognitive testing. This clinical assessment phase is particularly conducive to delivering PAC, given its requirement for informed consent from individuals suspected of being in the early stages of dementia to proceed with syndrome assessments.

### Study design

Reflexive Thematic Analysis (Braun & Clarke, 2022) was chosen for this study due to its adaptability and theoretical flexibility, making it ideal for exploring the complex nature of dementia diagnosis and PAC. This approach allows for a thorough examination of clinicians’ experiences and perspectives, encourages critical reflection on biases and assumptions, and enhances the rigour of findings (Campbell et al., 2021). It identifies and analyses themes within the data, uncovering the mechanisms and effectiveness of PAC in dementia care while integrating diverse perspectives for contextually grounded results (Braun & Clarke, 2022).

Semi-structured interviews were used for flexibility, allowing in-depth exploration of participants’ perspectives (Adeoye-Olatunde & Olenik, 2021). They facilitate open-ended dialogue, uncovering unexpected insights and themes (Wolff et al., 2019). They also help establish rapport and trust, encouraging frank and open dialogue (Blake et al., 2021). This approach captures rich, detailed data through interactive dialogue and follow-up questioning, ensuring a comprehensive understanding of participants’ experiences (Knott et al., 2022).

### Participants

A sample of clinicians trained to conduct PAC from the dementia service were invited to participate in the study, and participation was voluntary. Participants had to work within the Early Intervention for Dementia Service in the West Midlands, UK, and deliver PAC to people with dementia and their carers or families. A total of 10 clinicians participated in the study; 2 participants dropped out before the interview.

### Interview schedule

The second author is a former clinician of the EIDS (Early Intervention Dementia Service) team and used their knowledge of clinical practice to guide the development of interview questions. Whilst working within the service, they supervised a student who performed a service evaluation as part of their studies. The primary researcher used the findings from this study to construct meaningful questions to ask the clinicians. Then, the first author emailed the second author with their proposed schedule, and these were refined between them until finalised. The interview schedule was shared with the clinical team to determine if there was anything in particular they wanted to explore; however, none of the clinicians requested any changes. Clinicians were invited to participate in an interview; the interviews took a semi-structured approach. The researcher worked with a list of the information required from each respondent (Phillipowsky, 2020), with the wording and sequencing of questions tailored to fit individual participants.

These interviews allow for a qualitative approach exploring the issues in depth with clinicians' perspectives of what they felt the benefits of PAC were for people with dementia and their families, what challenges they experienced in implementing it, and anything they would change during the PAC process. The interviews followed an interview guide with 11 open-ended questions (see Table 1).

**Table 1.** Questions used during the semi-structured interviews held with clinicians.

Question order
What is your understanding of pre-assessment counselling? (*Prompt: How would you define pre-assessment counselling to someone?)*
What does pre-assessment counselling involve? (*Prompt: What does pre-assessment counselling consist of in your service? What should pre-assessment counselling involve? Describe your involvement with pre-assessment counselling in your role*
Could you tell me about the impact pre-assessment counselling has on a service user and their families? (*Prompt: What are the benefits to patients? What are the challenges to patients? Describe the experience patients typically have with pre-assessment counselling*
What impact does PAC have upon staff and services? *(Prompt: In the immediate sense? In the future?)*
Could you tell me about your role within this service? (*Prompt: what does your role involve? What kind of activities do you do in this role day to day? How long have you been working in the team for?*)
What would you say the signs of quality are in PAC? *(Prompt: What must PAC always include and why?)*
What are your views on whether PAC supports a timely diagnosis? *(Prompt: How does this differ from ‘quick diagnosis?’)*
How do you manage the different expectations or agendas within the PAC appointment? *(Prompt: What kind of expectations does the people with dementia tend to have? The carer tends to have? You tend to have?)*
How do you manage the balance of remaining sensitive to service user fears and anxieties whilst being open in dialogue? *(Prompt: Have there ever been times when this balance has been challenging to maintain? How did you bring the balance back?)*
What kind of training or guidance would all staff need to have delivered to ensure excellent PAC provision? (*Prompt: What universal or mandatory training must you do before you can deliver PAC? Do you have a handbook/framework you can refer back to?)*
Is there anything significant to you or anything we have not covered that you would like to add about your experience of pre-assessment counselling or anything that we have not covered that is important to you around the topic of pre-assessment counselling?

### Procedure

Nottingham Trent University and the Worcestershire and Herefordshire Health and Care NHS Trust obtained favourable ethical opinions for the study. Clinicians who expressed interest contacted the primary researcher, after which they received a participant information sheet and consent form. Upon returning the signed consent form, clinicians were provided with a copy of the topics to be discussed in the semi-structured interview in advance. This step aimed to ensure the appropriateness of the questions and allow the clinicians time to consider their responses.

Interviews were conducted via Microsoft Teams, following the trend of using video conferencing for qualitative interviews, which provides a convenient and accessible means for participants to engage, potentially widening participation (Lobe et al., 2020). They also foster familiarity and trust, enabling rich data collection despite physical distance (Nassaji, 2015), while saving time and resources by eliminating travel expenses, making them a cost-effective option for researchers (Khan & MacEachen, 2022). Each interview lasted approximately 60 minutes and was recorded using Microsoft Teams. Clinicians were encouraged to find a private and comfortable location for the interview to facilitate open and honest discussion. The primary researcher conducted the interviews between March and May 2023. Table 2 outlines how the primary researcher followed the analysis steps and the reflexivity and trustworthiness of reflexive thematic analysis.

**Table 2.** How the six steps of reflexive thematic analysis (Braun & Clarke, 2022) were implemented.

Steps	Description
Familiarising with the data	To familiarise themselves with the data, the first author read through the full transcripts and field notes, concurrently taking notes in chunks of critical reflexive comments
Generating initial codes	Thereafter, the first author started inductive coding for the transcripts
Generating themes	After four coding rounds, initial themes were developed with the first and third authors
Reviewing themes	This was an iterative back-and-forth process between codes and raw data until all authors collectively agreed on themes and, later, meaningful patterns. Thematic mapping was developed to schematise the relationships between the themes. As typical for reflexive thematic analysis, these maps were adapted several times before deciding upon a clear thematic structure
Defining and naming themes	The first, third, and fourth authors discussed summary themes and thematic maps, and they selected illustrative quotes
Producing the report	This collaboration eventually produced a full report with carefully selected quotes
Reflexivity and trustworthiness	The primary researcher’s positionality, as a practitioner experienced in dementia care, informed early engagement with the data, particularly in identifying systemic barriers and relational dynamics. Reflexivity was maintained through journaling, team discussions, and iterative interpretation, which challenged assumptions and strengthened analytic rigour. The diversity of co-authors’ perspectives supported critical reflection throughout

Please see Supplementary File 1 for the Reflexive Statement.

No demographic information was collected because clinicians were recruited within the same service, and anonymity may be compromised. Information sufficiency was achieved as clinicians provided rich and detailed accounts, with common themes emerging that adequately addressed the study’s aim and questions.

### Data analysis

The interviews were transcribed using NVIVO software and analysed using reflexive thematic analysis. This approach allowed for an in-depth exploration of clinicians’ experiences of PAC provision. The analysis was informed by a constructivist orientation which recognises that knowledge is shaped by individuals’ experiences and interpretations rather than objectively discovered. Positionality in qualitative research acknowledges the researcher’s background, values, and experiences, which can shape the research process, from data collection to interpretation (Berger, 2015). Reflexivity involves critically examining these influences to ensure transparency and awareness of potential biases (Finlay, 2002). In this study, the first author collected the data and was the primary analyst. With extensive experience working with people living with dementia in hospital and home settings, they brought an informed perspective on the challenges faced by people with dementia and carers in healthcare environments. Their understanding of PAC and engagement with healthcare professionals may have influenced the interpretation of quotes, particularly in recognising systemic barriers and the importance of tailored support. While this professional background provided contextual insight, reflexive practices were employed to mitigate potential biases. These included maintaining a reflexive journal, engaging in discussions with the research team to explore alternative interpretations, and prioritising participant narratives over preconceived assumptions. As this study formed part of a PhD programme, the wider research team contributed by reviewing the analysis and providing feedback to ensure clarity and coherence. To minimise potential bias, interviews were conducted using open-ended, neutral, and assumption-free questions, encouraging detailed responses, practising active listening, and preparing questions in advance.

## Findings

Three themes were identified through a rigorous coding and analysis process (see Table 3).

**Table 3.** Emergent themes and subthemes from analysis.

Theme	Subtheme
1. The person with dementia is central in their diagnosis journey	1a. Timely diagnosis is important for the wellbeing of people with dementia
	1b. Empowering people with dementia in the diagnostic journey
2. Candid conversations build strong therapeutic alliances	
3. People with dementia are more than their diagnoses	3a. Fostering resilience through comprehensive support
	3b. Living well with dementia

### Theme 1: The person with dementia is central in their diagnosis journey

Clinicians noted challenges like inconsistent person-centred practices and premature diagnostic pressure, where assessment and diagnoses are given before the person with dementia is ready. Person-centred care within a healthcare context prioritises the needs, preferences and values of the individual, enabling them to be actively involved with care planning and decision-making (Ekman et al., 2011). By emphasising a holistic approach, it ensures that care is individually tailored and not solely focused upon the medical condition (Santana et al., 2018). The person-centred care model facilitates a partnership between healthcare professionals and their patients which enhances satisfaction, autonomy and health outcomes (Kitson et al., 2013). Emphasising agency for people with dementia in timing and pace highlights the importance of person-centred approaches. PAC plays a vital role in the diagnosis journey as timely diagnoses respect autonomy, contrasting with rushed diagnoses that neglect needs and individual choices.

#### Subtheme 1a. Timely diagnosis is important for the wellbeing of people with dementia

Clinicians continuously discussed the role of PAC in facilitating diagnoses that prioritise the well-being of people with dementia through timely diagnoses as opposed to quick ones, where the former places the person with dementia’s interests at the forefront of healthcare. In contrast, the latter prioritises stakeholders’ interests.

“I think PAC definitely supports timely diagnoses, but I also think it depends on whether you are interested in what is best for the individual or what is best for the Trust! […] It's making sure that the person is ready to hear it in the right frame of mind. Essentially, timely diagnoses are person-centred. Quick diagnoses aren’t.” **Ash.**

Ash’s perspective underscores the significance of timely diagnoses in the context of PAC, highlighting how timely diagnoses prioritise the individual’s well-being, contrasting with rushed diagnoses that may overlook these considerations. This sets the stage for exploring the role of PAC in facilitating timely diagnoses and its impact on the emotional well-being of people with dementia. Charlie builds upon Ash’s emphasis on person-centred care by stressing the importance of working at the person with dementia’s pace, illustrating the practical implications of prioritising readiness for diagnosis.

“Working at their pace is quite important. You know, we always ask for a timely… timely diagnosis timely to me is when they're ready, not when we are. And… and I get that we have to diagnose people and move on and get all these rates up a do get that, but I think if we force in somebody through a system that's scared that's worried, doesn't understand it… they're gonna retract and we, we're not gonna engage. And it's fearful. It's horrible for them.” **Charlie.**

Morgan expands upon Charlie’s insights by acknowledging mistakes and the potential harm caused by rushing diagnoses.

“And you know, we made mistakes. […] we've encouraged people to go along with it when perhaps they weren't ready. And I think we did harm to people. You know, you can do harm to people.” **Morgan**

Morgan’s perspective prompts a critical examination of past practices. It highlights the need for a person-centred approach in PAC to ensure that diagnoses are delivered at the right time, with sensitivity to the individual’s emotional state. Jules’s approach to supporting decision-making after initial assessment resonates with Morgan’s reflection, demonstrating a concrete example of how clinicians can mitigate harm and promote well-being through person-centred practices in PAC.

“Yeah, I had a lady who sort of said she wanted to move forward with assessment. Then she got quite upset at the end of PAC and the family did want it to move forward with assessment. And I just gave time, and I did explain, you know, the benefits of the early diagnosis again at the end of the not, you know, at the end of my actual initial assessment. And… And I gave her some time and I always say to people it's not even a decision you got to make now. I can go away and give you a phone call or I can come back out and see you in a week’s time. You can have a bit of time to digest what I've said, and I could go through things again with you.” **Jules**

Jules's emphasis on providing people with dementia with the opportunity to digest information and make decisions at their own pace underscores the role of PAC in facilitating a more empowering and supportive diagnostic experience for people with dementia.

In summary, this subtheme highlights the vital role of sensitive delivery and prioritising the well-being of people with dementia in PAC. It’s a crucial tool for timely diagnoses that meet the needs of people with dementia, contrasting with quick diagnoses that may neglect these considerations. Clinicians stress the importance of distinguishing between timely and rushed diagnoses, with the former highlighting person-centred care, reducing errors, and ensuring diagnoses are delivered at the right time.

#### Subtheme 1b. Empowering people with dementia in the diagnostic journey

Clinicians noted PAC’s role in empowering people with dementia to control their diagnosis journey, highlighting the importance of agency. PAC allows people with dementia to make informed decisions at their own pace, enhancing well-being by restoring their sense of control during the diagnostic journey.

“I think having that choice is really important because, you know, especially with something like dementia, you know, often people aren't able to make choices in the later stages. So, giving people that power, giving people that sense of control and return that agency to them is really important, really, really important.” **Ash**

Ash’s interpretation emphasises the ethical imperative of empowering people with dementia in decision-making processes, particularly in contexts where cognitive decline may limit their ability to exercise autonomy.

“So yeah, I'll reiterate the importance of this being patient choice. There's no right or wrong decision. It's their decision. And you know, and ‘I can walk out this door today [PAC is typically delivered in people with dementia’s homes] and if you don't want to see me again, then that's absolutely fine’.” **Morgan**

Morgan explains the importance of respecting individual preferences and fostering a supportive environment for decision-making by acknowledging the person with dementia’s right to refuse assessments and offering reassurance regarding the freedom to discontinue PAC. Morgan’s emphasis on choice supports Charlie’s perspective, which exemplifies the practical application of autonomy principles by prioritising the wishes of the person with dementia over external influences.

“And I just have to be honest with the family. ‘I'm really sorry, but this is mum's assessment. And I'll take your points. They are very valid. I appreciate your worries, but this is about mum and what she wants’.” **Charlie**

Charlie highlights the need for clear communication and boundary-setting in PAC, ensuring that decisions are guided by the individual’s preferences rather than external pressures. Similarly, the importance of providing comprehensive information to enable informed decision-making by people with dementia and families is highlighted:

“…they can then make an informed decision about whether they want to move forward or not. They can't make that informed decision if you've not given them all the information.” **Jules**

Providing comprehensive information not only enabled informed decision-making, but further reinforced the sense of autonomy. By recognising the role of clinicians in facilitating understanding and autonomy in people with dementia through transparent communication, Jules underscores the ethical imperative of empowering people with dementia to partake in the decision making.

Empowering people with dementia to decide the timing and pace of their diagnostic journey fosters open dialogue. Clinicians reassure them that assessment choices are theirs, creating a safe space for discussing fears and cognitive changes. Many people with dementia welcome discussing this with family and those who support them. These exchanges build trust, dignity, and self-determination in the person with dementia-professional relationship, equipping people with dementia with knowledge and agency for personalised dementia care.

### Theme 2: Candid conversations build strong therapeutic alliances

The paramount importance of candid conversations as the cornerstone of PAC appointments between clinicians, people with dementia and their carers was repeatedly noted. Every participant emphasised the critical role of honesty and openness in these interactions, highlighting its integral nature in fostering trust and building strong alliances.

“Definitely people trust you more as well. There's less of that kind of guarded feeling, and yeah, I just think it's better all round for everybody. People feel that they can tell you more because you've been up front with them. I just I think it works really well.” **Ash**

Ash’s insight underscores that transparency in PAC fosters trust and builds strong therapeutic alliances between healthcare professionals, people with dementia, and their carers. Ash’s focus on building trust through honesty and openness is akin to Kit’s perspective, which further emphasises the importance of transparency in communication, particularly in managing expectations and addressing stigma.

‘…you have to be very clear. You have to let people know what it is about, and you cannot just sugar coat it or… or whatever, because that's misleading the person and disrespectful. The fact that we've got the word dementia in our title [Early Intervention Dementia Service], I think really helps.’ **Kit**

By using clear language and avoiding euphemisms, healthcare professionals aimed to provide individuals with realistic expectations and prepare them for the diagnostic journey ahead, promoting informed decision-making and reducing anxiety. Stacy also highlights the role of honest and transparent conversations in creating a supportive environment for discussing dementia diagnosis.

“You’re starting off a process by being very open, honest, transparent about the purpose of… of the appointment when you when you go out and see somebody. There's no kind of hiding or talking in terms of memory problems or, um, cognitive difficulties or other terms that that we sometimes use. I think it's just an… an openness really, about kind of opening that conversation around dementia and your patients are kinda given the floor to discuss their views, their thoughts and feelings.” **Stacy**

Clinicians aim to inform, support, and establish a partnership with people with dementia and their carers, ultimately enhancing the quality of care provided. Jules agrees with this but mentions how the influence of media and internet searches can impact peoples understanding of dementia and the importance of expectations.

“I think some, you know, some people have Googled the thing to death, haven't they? Before you've gone to Doctor Google, which has a lot to answer for. So, and especially with the new medications being spoken about recently on… on you know, on the television, that it’s some magic cure, and they think that things are gonna be sort of ready to be prescribed within, you know, a month or so. And we have to be honest about that, that it's actually not and that it doesn't work for everybody and it's not for every type of dementia. **Jules**

Jules highlights the importance of addressing misconceptions and managing expectations in PAC. Jules’s emphasis on combating stigma and promoting transparency is echoed by Morgan, who underscores the importance of using clear language and advocating for openness of the diagnostic process.

“I think when that when the patient receives our letter and then our… our title is at the top, I think that's also at the start of things. You know, it's that using that word, you know. By not using it, I think we're almost colluding with the stigma around it. Let's get it out there, let's be open, let's be honest, right, right from the beginning, before we even walk through the door about what… what we're about what we're doing.” **Morgan**

Using clear language such as “dementia” in communication materials, healthcare professionals advocate for transparency and honesty, fostering a supportive environment for people with dementia and families throughout the diagnostic journey.

These findings highlight how candid conversations are vital in PAC appointments and benefit the triadic relationship of the person with dementia, carer and clinician. These discussions foster trust, equip individuals with realistic expectations, and address stigma, ultimately empowering them for the diagnostic journey. Honest communication is crucial in improving care and supporting people with dementia and their carers throughout the process.

### Theme 3: People with dementia learn to accept that a diagnosis does not define them or their lives

Clinicians noted that the PAC appointments serve as a pivotal moment for empowering people with dementia and families with knowledge and strategies to navigate the challenges of dementia while optimising their QoL and preserving their sense of identity and purpose.

#### Subtheme 3a. Fostering resilience through comprehensive support

The PAC appointment is crucial for providing people with dementia and their carers with essential information and dispelling misconceptions, highlighting that a dementia diagnosis is not the end but the start of a journey with support and appropriate resources available.

“…quality of life is a little bit better when you know what you're dealing with. Um, and in particular for families knowing what support’s out there and when to get it and who to get it from. And you know, because there’s things out there for them. Not just talk to Doctor Google.” **Jules**

In Jules’s perspective, the value of PAC in enhancing QoL becomes evident. By equipping individuals and families with comprehensive information about dementia and available support services, PAC empowers them to navigate their diagnosis confidently. This highlights the importance of PAC in alleviating fears and improving QoL for people with dementia and carers. Similarly, Jan emphasises the importance of understanding individual needs and preferences to tailor support, thus deepening the discussion on the collaborative nature of PAC discussions.

“You’ve got to take the time to understand the person, what their needs are really and finding out from their point of view what they would like to happen. [Also], what support they think they need, what's within your remit, [and] what you're able to do. And if you're not [able to do something] who they can contact to be able to gain that information.” **Jan**

By tailoring support services to meet these needs, clinicians facilitate informed decision-making and enhance QoL for people with dementia and their carers. Jan’s perspective on the collaborative nature of PAC discussions is supported by Ash, who emphasises the importance of ongoing support and dialogue beyond the initial session. Ash underscores the role of PAC in the care continuum for people with dementia and their carers, thus deepening the discussion on the long-term impact of PAC.

“And we’ll say, you know, we've got lots of resources, lots of organisational support in the local area for you and you know it's about living your life as well so if things do change then you know where to go for support and everybody has an allocated OT [occupational therapist] or nurse, so there's always somebody that you can contact to talk to.” **Ash**

While PAC serves as an essential step in providing knowledge and resources, ongoing support is vital for preserving QoL in the long term. Morgan emphasises the benefits of proactive management in improving future outcomes for people with dementia and their families, deepening the discussion on the importance of early detection and comprehensive support.

“I'll say something along the lines of… and, you know, the feeling nowadays is the earlier that you intervene with conditions like the dementia, the better your future can be. Forewarned is forearmed. You know, if you know early on that there's something changing, then it can give you and your family the opportunity to understand it, to learn about it.**” Morgan.**

In summary, the PAC appointment is vital for providing people with dementia and their carers with essential information and support, dispelling misconceptions about dementia and instilling hope. Clinicians stress the importance of comprehensive information and ongoing dialogue to alleviate fears and improve QoL. PAC discussions aim to tailor support to individual needs, empowering confidence in diagnosis navigation. Ultimately, PAC is critical in the care continuum, facilitating informed decisions and supporting sustained QoL.

#### Subtheme 3b. People with dementia understand that it is possible to live well with dementia and retain lifestyle

Stigma and misconceptions about dementia contribute to fear. In PAC, clinicians emphasise that it is possible to live well with dementia and ease anxieties surrounding that concept. They highlight that people with dementia can maintain lifestyles with support, empowering them to engage in activities and enhance well-being.

“So, it… it's talking to people about that… there's lots of people that live very well with dementia and, you know, still drive still, you know, and carry on with activities, still enjoy life and you know good relationships. And so, I think it's you know highlighting that to people, yeah. […] You talk to them about the support, the interventions that can be put in place and… and, you know, support people to live really well.” **Jamie**

Jamie highlights that many individuals with dementia continue to lead fulfilling lives, underscoring the need to balance discussions about challenges with opportunities for support and intervention. Kit elaborates on the role of PAC in promoting realistic expectations while offering hope for individuals and families facing a dementia diagnosis.

“We inform patients about the service to increase their knowledge about dementia. To start the conversation about the interventions they can receive, which way may improve the quality of life with diagnosis of dementia, the support they and their family can receive. And I suppose, the best of it is that… kind of… I'm not saying you can have a positive outlook from pre assessment counselling but almost to have a little ray of hope that perhaps the situation may not be as bad as they imagined.” **Kit**

By informing people with dementia about available interventions and support services, Kit aims to instil optimism without downplaying the challenges ahead. Part of the adjustment work is acknowledging fears, concerns, and distress, giving space for this to be heard and processed, and balancing this with realistic information about what to expect and what is available to support QoL, agency, and identity. This allows people with dementia to build hope and facilitate a sense of control within the realities of living with dementia. Pat underscores the importance of preserving a sense of normalcy and optimism through practical strategies.

“The ultimate is to make things better, you know, and to hope that what comes out is the quality of life stays the same and it doesn't dip and it doesn't. Yeah, okay, there might be the diagnosed dementia, but we can live well with it.” **Pat**

Pat’s perspective on the abundance of resources available for supporting individuals with dementia leads to Ash’s insights, reinforcing the message of hope and empowerment by acknowledging challenges while highlighting the availability of practical strategies and support resources.

“We talk about how it is possible to live well with dementia too even though it may come with challenges. However, there is lots of practical strategies and lots and lots of resources to live well with dementia. Lots of resources for onward support.” **Ash**

Stigma and misconceptions create fear for people with dementia, but PAC lets clinicians address these beliefs, conveying that living well with dementia is possible. Through PAC, clinicians offer interventions and support, reducing fear and fostering hope while empowering people with dementia and their families with knowledge and strategies to navigate dementia’s challenges and maintain fulfilling lives.

## Discussion

This study, aimed at exploring the significance of PAC for people with dementia, is the first to explore the perspectives of clinicians relating to the provision of PAC with people with dementia and their caregivers. The study provides evidence why PAC is effective in supporting timely diagnoses, arguing the benefits of its implementation to all involved within the triadic relationship. Findings were conceptualised into three themes: (1) The person with dementia is central in their diagnosis journey; (2) Candid conversations build strong therapeutic alliances; (3) People with dementia are more than their diagnoses (see Table 3).

The first theme noted that people with dementia are central in their diagnosis journey. Specifically, that timely diagnosis is important for the well-being of people with dementia (Prince et al., 2011) and that people with dementia are empowered in the diagnostic journey (Merl et al., 2022). Clinicians emphasised the importance of timely diagnoses for the well-being of people with dementia yet recognised the nuanced nature of diagnosis delivery. While quick diagnoses may serve statistical goals, clinicians emphasise the need for person-centred, careful, and collaborative approaches, acknowledging the conflict between driving faster diagnostic rates and ensuring patient welfare. Quick diagnoses may result from plans by the NHS Commissioning Board to reward practices for assessing people with dementia who may be showing early signs of the condition (NHS Commissioning Board, 2013). Moreover, the findings underscored the multifaceted impact of diagnosis, highlighting clinicians’ need to assess its potential effects on the overall well-being and engagement of people with dementia in post-diagnostic services. The tensions between the interests of commissioners, service users, and the ethical imperative to prioritise the welfare of people with dementia were palpable, suggesting a delicate balance in PAC delivery.

The findings emphasised the importance of empowering people with dementia in decision-making processes surrounding diagnosis; acknowledging and restoring agency to people with dementia, particularly in the early stages of dementia, to facilitate informed decision-making and improve overall healthcare outcomes (Street et al., 2012). Moreover, the study underscored the imperativeness of creating spaces for people with dementia to express their preferences, needs, and perceptions, both privately and publicly, to ensure their voices are heard in decision-making processes (Fetherstonhaugh et al., 2013; Tyrrell et al., 2006). By adopting a person-centred approach and recognising the capacity of people with dementia to make decisions, clinicians aimed to alleviate pressures, promote autonomy, and enhance communication between caregivers and providers, ultimately striving for optimal outcomes in the diagnostic journey.

The second theme found that candid conversations build strong therapeutic alliances. Clinicians highlighted the necessity of openness, honesty, and transparency during PAC appointments to establish trust and rapport. Despite the discomfort associated with discussing ‘the D word,’ clinicians recognised the harmful impact of stigma and the ambiguity of euphemisms in the diagnostic process (Bamford et al., 2004; Kaduszkiewicz et al., 2008). Through candid dialogue, nurses aimed to dispel misconceptions and address the origins of fear, stigma, and anxieties surrounding dementia, acknowledging the validity of the feelings and experiences of people with dementia. Encounters with dementia, through familial ties or vulnerability (Corner & Bond, 2004), were identified as influencers of people with dementia’s perceptions and concerns. By engaging in honest conversations that validate people with dementia’s experiences and dispel myths, clinicians aim to build trust and increase diagnostic rates, recognising the role of addressing stigma in facilitating timely diagnoses. Despite the sensitivity required, clinicians emphasised the importance of frank discussions in fostering trust and dismantling stigma, leading to improved diagnostic outcomes and enhanced engagement of people with dementia in dementia care.

The third theme found that people with dementia are more than their diagnoses. Specifically, importance was placed on fostering resilience through comprehensive support and cultivating an understanding of living well with dementia (Whelan et al., 2020). Clinicians emphasise the role of PAC in fostering resilience through comprehensive support, which includes clear communication and addressing misconceptions and stigma surrounding dementia (Bamford et al., 2004; Kaduszkiewicz et al., 2008). Acknowledging the challenges posed by internet searches for health information, clinicians stressed the importance of providing accurate and tailored information during PAC appointments to facilitate informed decision-making. Additionally, the PAC appointment serves as a platform for understanding the individual needs of people with dementia and their families, enabling referrals to supporting services across healthcare settings and to tailor the assessment in terms of who they would like involved, level of information provided, and who the diagnosis is shared with.

Furthermore, the PAC appointment aids in informing people with dementia and their families of the realities of dementia, emphasising that a diagnosis does not signify the end of life but rather access to support and interventions can enable people to live as well as possible. Nurses underscored the importance of honesty regarding dementia progression while emphasising the potential for engagement in enjoyable activities. By dispelling misconceptions and reducing stigma, PAC empowers people with dementia to maintain their QoL and engage in activities they enjoy. Many people with dementia report a decline in QoL, which is often about poor support, services, and stigma (Ferdous, 2020; Mate et al., 2012; Stites et al., 2017), as evidenced by research indicating that people with dementia do not perceive a decline in quality-of-life following diagnosis. This holistic approach to support and decision-making throughout the diagnostic journey emphasises the importance of empowering people with dementia to lead fulfilling lives beyond their diagnosis.

### Clinical implications

There appears to be a divide between commissioners and practitioners as to what a timely diagnosis actually is versus what a timely diagnosis means (c.f., Dhedhi et al., 2014). Based on the present study’s findings, clinicians must be given time to communicate information, support, and personalised care within the PAC appointment while ensuring that people with dementia and carers’ needs are addressed. All parties within the triadic relationship benefit, but none more so than the person with dementia (La Fontaine et al., 2014). The findings demonstrate that PAC is associated with better outcomes. Specifically, PAC allows for comprehensive and personalised care that addresses the unique needs of people with dementia and their carers, fostering a more supportive and understanding environment.

Despite PAC’s demonstrated effectiveness, such appointments are not consistently available across NHS Trusts. There is a pressing need for the consistent implementation of dedicated PAC appointments to ensure all people with dementia can benefit from timely diagnoses and comprehensive care. The establishment of these appointments should be prioritised and recognised for their role in improving outcomes for people with dementia.

### Strengths and limitations

This study utilised qualitative methodology to prioritise clinicians’ perspectives, discussing concepts that enhanced the depth of the data as opposed to quantitative research. The study recruited from a multidisciplinary team of psychologists, advanced care practitioners, nurses, and occupational therapists, all whom specialise in the care of people with dementia, facilitating a deeper contextualised understanding of the study’s findings. Although this study obtained enough richness in the current data, future research would benefit from a larger and more diverse sample which may address any sample-related bias this study may have had. The research has been able to understand the psychological implications of potentially receiving a diagnosis of dementia and how these are sensitively mitigated through the people who support people with dementia and carers during this process.

PAC primarily supports individuals who are actively seeking an assessment and have the capacity to engage in decision-making. Those lacking insight or further cognitive decline are typically referred to older adult community mental health teams for assessment in their best interests. While this model facilitates informed choice, it may not fully address the needs of individuals who never perceive themselves as ‘ready’ for diagnosis, highlighting an ongoing challenge in dementia care.

The recruitment strategy may have attracted participants with strong views on PAC, potentially influencing the findings. Conducting interviews via Microsoft Teams provided clinicians with flexibility in timing and setting. However, online data collection may limit response depth, contextual richness, and participant diversity by excluding those with limited technical skills. Demographic data was not collected due to the recruitment from a single NHS Trust, which could lead to potential identification. This study highlights the need for further research in diverse cultural contexts, as perceptions of dementia and care provision vary significantly across countries (Brooke et al., 2018; Gove et al., 2021). Understanding how cultural factors influence PAC delivery is crucial, especially in settings with different healthcare funding models and varying access to early dementia services. While PAC is designed to be inclusive and adaptable to individuals from diverse linguistic and cultural backgrounds, access and engagement may vary. The service accommodates non-English speakers through interpreters and adjusts discussions to align with cultural perspectives on dementia. However, as with many healthcare services, there remain challenges in reaching underrepresented groups, and referrals from some cultural communities continue to be lower than expected. Expanding this research globally could provide critical insights into how PAC can be effectively implemented across different healthcare systems.

## Supplemental Material

Supplemental Material - Understanding the essential components and effectiveness of pre-assessment counselling (PAC) in providing a timely diagnosis according to NHS clinicians Supplemental Material for Understanding the essential components and effectiveness of pre-assessment counselling (PAC) in providing a timely diagnosis according to NHS clinicians by Marie Janes, Anna Buckell, Bethany A Jones, Miriam Sang-Ah Park and Stephen P Badham in Dementia

## Data Availability

Data sharing is not applicable to this article as no datasets were generated or analysed during the current study. *
