# Supplementary material for: Understanding the essential components and effectiveness of pre-assessment counselling (PAC) in providing a timely diagnosis according to NHS clinicians
Source: Dementia (London). 2025 May 26;25(2):443–61. doi: 10.1177/14713012251345928 (PMC12816407; doi:10.1177/14713012251345928)
Supplement: Supplemental Material - Understanding the essential components and effectiveness of pre-assessment counselling (PAC) in providing a timely diagnosis according to NHS clinicians [file sj-pdf-1-dem-10.1177_14713012251345928.pdf]

Reflexive Statement

This study is part of a broader project aimed at comprehensively understanding the impact of PAC within the journey of a diagnosis of dementia, a subject area necessitating nuanced exploration. The setup of this study benefited from collaborative efforts, notably with the second author, who previously worked within the team from which HCPs were recruited. This prior association likely facilitated participant recruitment, underscoring the nuanced role of pre-existing relationships in research settings. Additionally, the first author's background in working within a memory clinic environment lent a depth of understanding to the study's context. Recognising that these shared identities between researchers and participants may have subtly influenced the formulation of interview questions and subsequent data analysis is essential. However, it is worth noting that the dataset yielded rich, open, and in-depth responses across the board.

To maintain transparency and cultivate self-awareness throughout the research process, the primary author maintained a reflexive journal in which interviews took place and generated themes. This journal served as a repository for reflections on how personal values, positions, and privileges might have influenced interpretations of the research findings. Given the inherently emotive nature of dementia, the first author's extensive experience in working with individuals undergoing assessments and those recently diagnosed, along with their carers, inevitably shaped both the data interpretation and thematic formulation.

Recognising the ethos of continuous improvement and reflective practice, it is essential to acknowledge a significant oversight. Some participants expressed nervousness upon receiving Microsoft Teams invites titled "pre-assessment counselling interview", evoking associations with formal job interviews. Upon reflection, such terminology may have inadvertently induced unnecessary tension, at least before establishing rapport and settling into the conversation. Opting for the term 'discussion' instead of 'interview' would have been more appropriate. This underscores the importance of sensitivity in communication and the need for ongoing reflexivity to mitigate unintended consequences.

Regular discussions with the broader research team enriched the analytical process throughout the data analysis phase. Leveraging their extensive expertise in qualitative research methods, particularly reflexive thematic analysis, contributed to rigour and ensured diverse perspectives.
